# Supplementary material for: Anti-Influenza Virus Study of Composite Material with MIL-101(Fe)-Adsorbed Favipiravir
Source: Molecules. 2022 Mar 31;27(7):2288. doi: 10.3390/molecules27072288 (PMC9000774; doi:10.3390/molecules27072288)
Supplement: Supplementary file 1 [file molecules-27-02288-s001.zip › molecules-1619102-supplementary.pdf]

Supplementary Materials

Anti-Influenza Virus Study of Composite Material with MIL-101(Fe)-Adsorbed Favipiravir

Mengyuan Xu, Xi Li, Huiying Zheng, Jiehan Chen, Xiaohua Ye and Tiantian Liu *

School of Public Health, Guangdong Pharmaceutical University, Guangzhou 510310, China; 18762176339@163.com (M.X.); lixi8813@163.com (X.L.); zhyzhy_2021@163.com (H.Z.); chenjie.han@163.com (J.C.); smalltomato@163.com (X.Y.)

* Correspondence: liutiantian1212@126.com

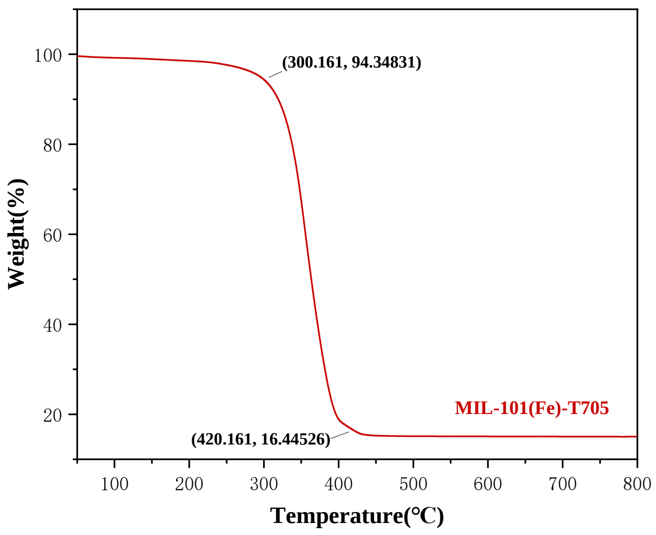

Figure S1. TGA curves of MIL-101(Fe)-T705.

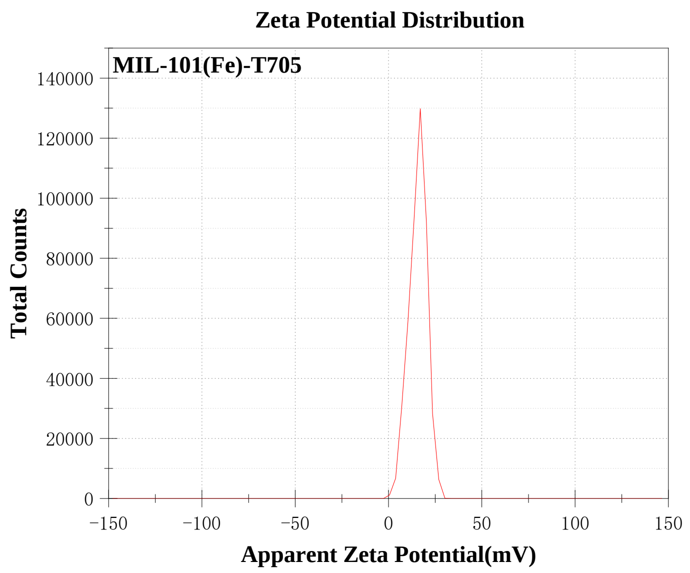

Figure S2. Zeta potential distribution of MIL-101(Fe)-705.

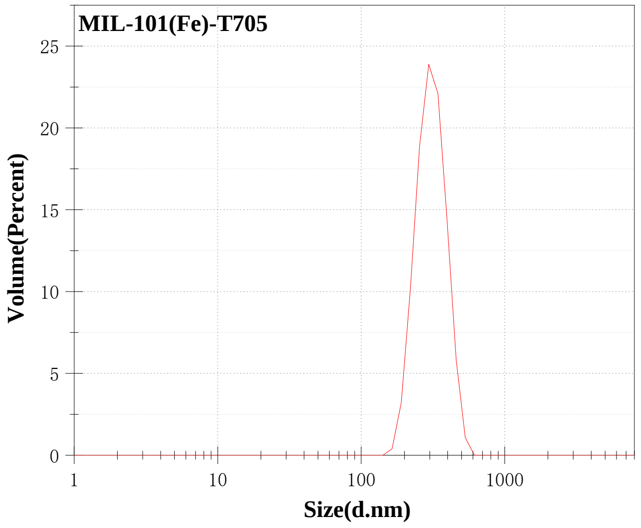

Figure S3. Particle size distribution of MIL-101(Fe)-T705.

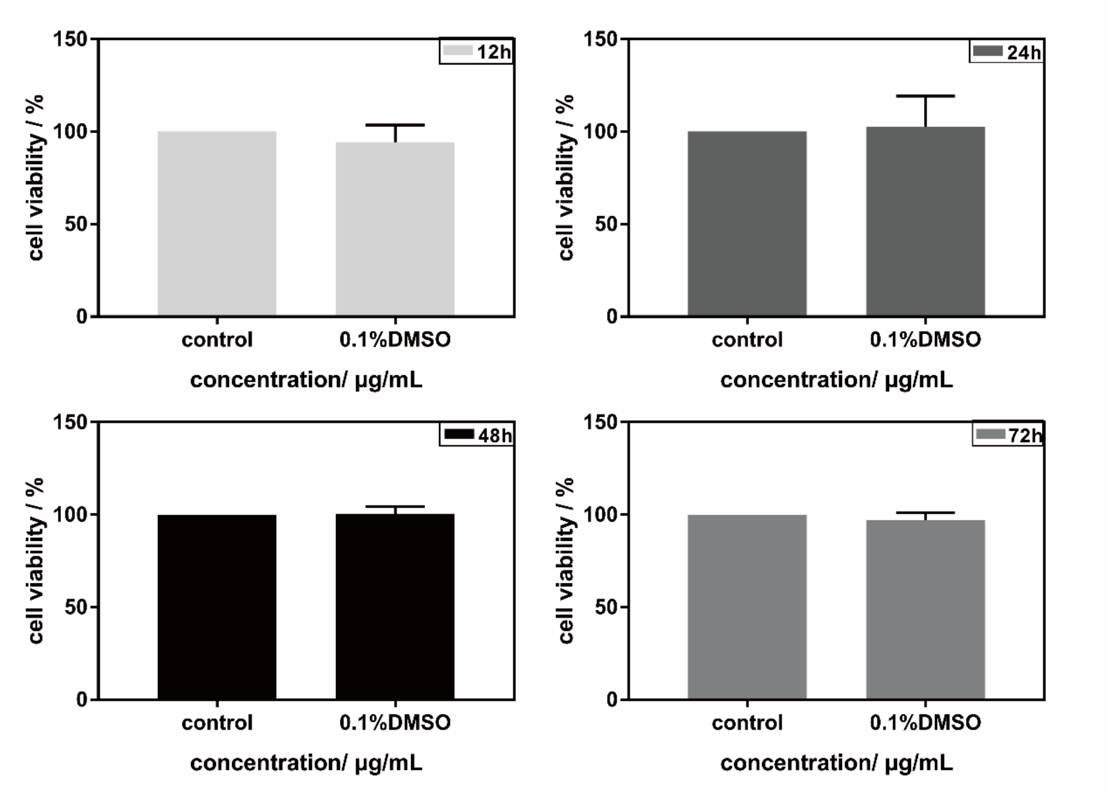

Figure S4. Cytotoxicity of MDCK cells with 0.1%DMSO after 12h, 24h, 48h, 72h.

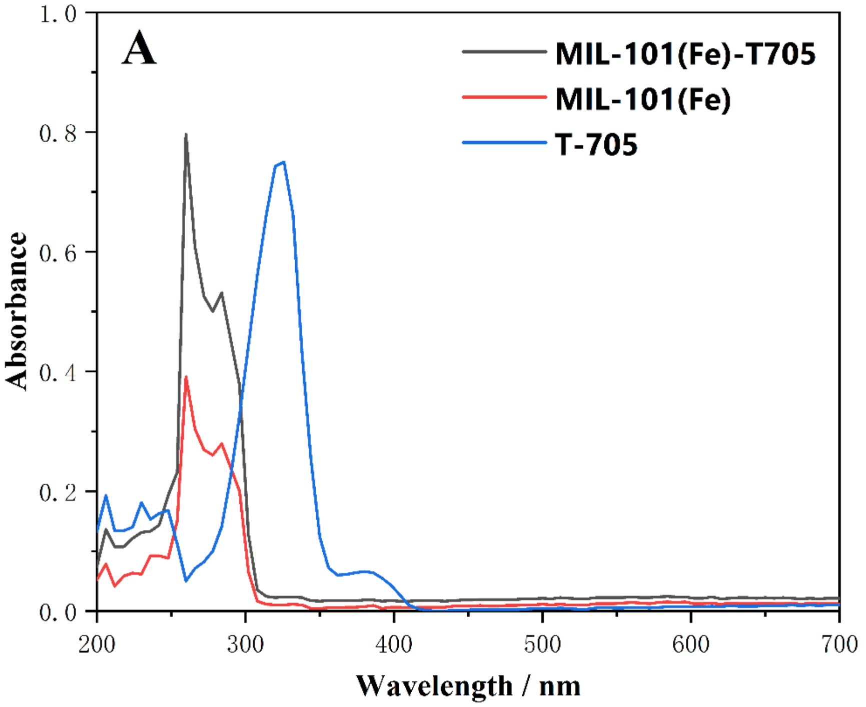

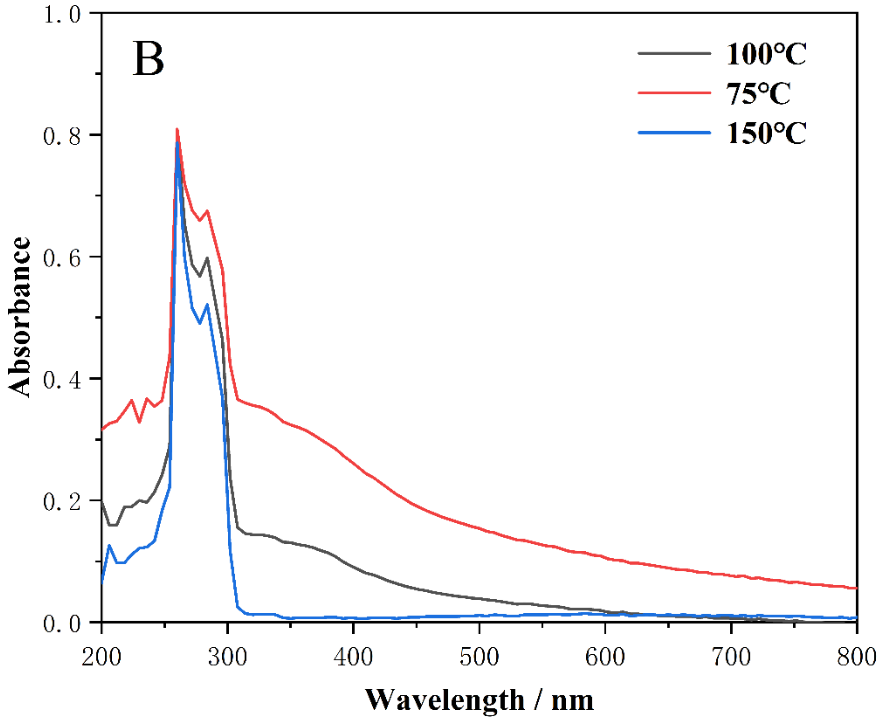

B
100°C
75°C
150°C
Absorbance
Wavelength / nm
1. 0
0. 8
0. 6
0. 4
0. 2
0. 0
200
300
400
500
600
700
800

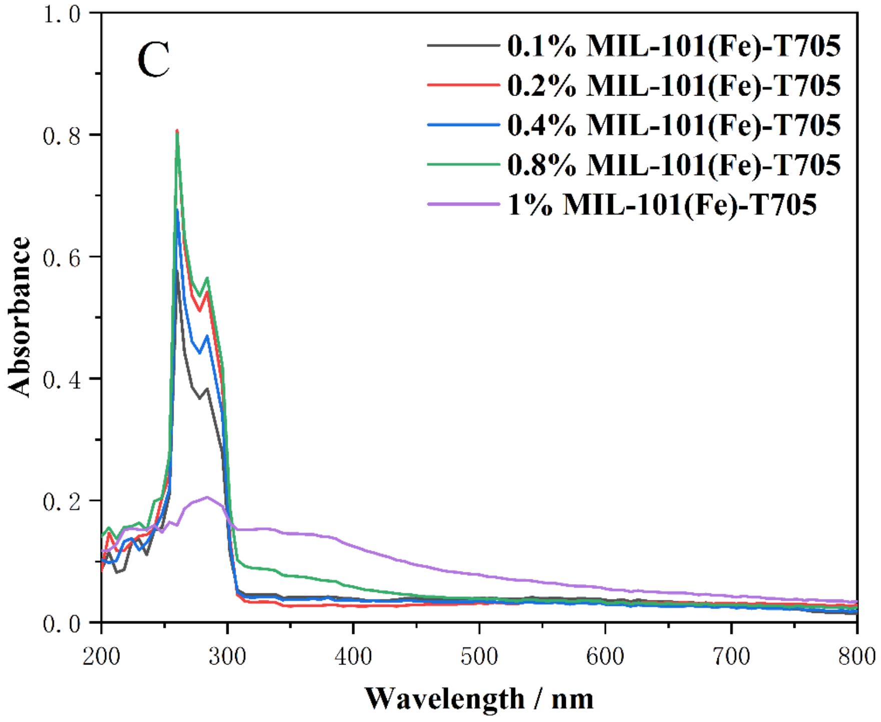

C
0.1% MIL-101(Fe)-T705
0.2% MIL-101(Fe)-T705
0.4% MIL-101(Fe)-T705
0.8% MIL-101(Fe)-T705
1% MIL-101(Fe)-T705
Absorbance
Wavelength / nm
1. 0
0. 8
0. 6
0. 4
0. 2
0. 0
200
300
400
500
600
700
800

Figure S5. UV-Vis absorption spectra of fabricated sample (A) MIL-101(Fe), MIL-101(Fe)-T705 and T-705. (B) T-705 at different reaction temperature. (C) Different amounts of T-705 are called 0.1% MIL-101(Fe)-T705, 0.2% MIL-101(Fe)-T705, 0.4% MIL-101(Fe)-T705, 0.8% MIL-101(Fe)-T705, 1% MIL-101(Fe)-T705.

Table S1. RT-PCR reaction system.

Reagents	Dosage（μL）
PCR mixture	20μL
Taq DNA Polymerase	0.2μL
Reverse transcriptase	0.5μL
Termplate	5μL
Total	25μL

The reaction conditions of the system: 5min reaction at 48°C, 2min reaction at 94°C, [10s reaction at 94°C, 35s reaction at 55°C] This process was cycled 40 times, fluorescence was collected, and finally solubility curve analysis was performed to ensure that the PCR product was a single specific target fragment. All samples were reacted in parallel for 3 wells, and the Ct (Threshold cycle) values of the samples were detected, and the experimental results were used to calculate the relative expression levels of genes by $2^{-\triangle\triangle Ct}$.
